# Application of the pressure cooker technique for transarterial embolization of brain arteriovenous malformations: Factors affecting obliteration and outcomes

**DOI:** 10.3389/fneur.2023.1133091

**Published:** 2023-04-13

**Authors:** Dan Lu, Yuqian Li, Zijian Yang, Zhenwei Zhao, Wei Fang, Lei Chen, Tao Ma, Naibing Wang, Xueliang Li, Tao Zhang, Jianping Deng

**Affiliations:** ^1^Department of Neurosurgery, Xi’an International Medical Center Hospital, Xi’an, China; ^2^Department of Neurosurgery, Tangdu Hospital, Air Force Medical University, Xi'an, China

**Keywords:** brain arteriovenous malformation, embolization, complication, pressure cooker technique, Onyx

## Abstract

**Objective:**

The typical pressure cooker technique (PCT) and several modifications with similar mechanisms have been introduced to enhance the embolization of brain arteriovenous malformations (bAVMs). This study aimed to assess the effectiveness of transarterial embolization of bAVMs with the PCT.

**Method:**

From January 2019 to December 2021, 125 consecutive patients with bAVM managed by transarterial embolization in the prospective database on cerebral vascular diseases of a single center were retrospectively reviewed. Patient data and lesion characteristics were collected. According to the treatment strategy, the patients were assigned to the PCT group (46 patients) and conventional embolization technique (CET) group (79 patients).

**Results:**

Baseline patient features were comparable between the two groups. After the first procedure, complete obliteration immediately was observed in 61 and 42% of patients in the PCT and CET groups, respectively. The rate was markedly elevated in the PCT group (*p* = 0.04). In subgroup analysis, the rate of immediate complete obliteration was starkly increased in PCT group patients with Spetzler-Martin grade I/II bAVM (86 and 53% in the PCT and CET groups, respectively; *p* = 0.0036). The overall complication rates were similar in the two groups (13 and 10% in the PCT and CET groups, respectively; *p* = 0.77). In multivariable analysis, nidus size >3 cm (OR = 8.826, 95% CI: 1.250–62.312; *p* = 0.03) and deep location (OR = 8.576, 95% CI: 1.480–49.690; *p* = 0.02) were significant factors affecting complete obliteration in the PCT group.

**Conclusion:**

The PCT may yield a higher rate of immediate complete obliteration with transarterial embolization of bAVMs, without increasing the rate of procedure-related complications.

## Introduction

Brain arteriovenous malformations (bAVMs) are rare and complex vascular lesions with an overall annual hemorrhage risk of 2.3%; however, different yearly odds of hemorrhage were reported for bAVMs unruptured (1.3%) and ruptured (4.8%) at first diagnosis (1). The ultimate therapeutic goal in bAVMs is total nidus and arteriovenous shunt suppression. Incomplete nidal obliteration results in no hemorrhage risk reduction (2).

In the last 10 years, embolization techniques have been transformed by the emergence of the novel liquid embolic agent ethylene vinyl alcohol copolymer (Onyx, ev3). Onyx represents a non-adhesive agent with prolonged polymerization time, resulting in improved nidus penetration with slower filling (3, 4). Onyx changed the situation of too low cure rate after embolization, and complete cure has been achieved with Onyx embolization alone in many cases (5, 6). The standard injection allows Onyx to revolve around the microcatheter’s tip to generate a solid plug. Following plug formation, Onyx advances anterograde into the nidus of bAVMs (7). However, such plug sometimes has unpredictable effects: Onyx advances forward and emerges with excessively high retrograde flow, occluding the proximal normal vessel or increasing the risk of vessel rupture during microcatheter withdrawal.

The pressure cooker technique (PCT), first developed by Chapot et al. to generate an anti-reflux plug of coils and glue, enables a continuous Onyx injection into the nidus while steering clear of unacceptable reflux, thereby improving the rate of curative embolization (8). Similar techniques with some modifications, which have the same mechanisms, have been introduced by several physicians. However, all available reports had small sample sizes (9–11). In the present work, we retrospectively assessed bAVM cases treated with the PCT and compared these results with those of other patients with similar bAVMs administered the conventional embolization technique (CET). The purpose was to examine the effectiveness of the PCT and possible factors related to angiographic and clinical outcomes.

## Materials and methods

### Patient population

The prospective database on cerebral vascular diseases of the Neurosurgery Department in Tangdu Hospital was retrospectively analyzed. From January 2019 to December 2021, 125 consecutive patients with bAVMs treated by endovascular embolization were included. All patients were embolized exclusively with Onyx 18 in an attempt to achieve complete occlusion of the nidus in the first procedure. The patients’ demographic features, e.g., age and sex, were assessed. Angioarchitectural parameters for bAVMs, including size, diffuseness, location, draining veins, occurrence of perforating arterial feeders, hemorrhage, Spetzler-Martin grade, and presence of pial or dural arteriovenous fistulas (AVF), were recorded according to preoperative digital subtraction angiography (DSA), traditional MRI or CT data. According to the treatment strategy, we divided the patients into PCT group and CET group, and then compared the different results after the first procedure between the two groups. All examinations were carried out by the same group of experienced interventional neuroradiologists and the staff. The present study had approval from the institutional review board.

Modified Rankin Scale (mRS) score assessment was carried out by a neurologist and/or an experienced clinical research nurse, with no involvement of the treating neurosurgeons. The pre-treatment functional status was evaluated during pre-treatment clinical visits or at admission; follow-up data were obtained during post-treatment clinical visits, at additional hospital admissions, or by phone. Angiographic follow-up by DSA was scheduled 1 year posttreatment.

### Procedure and the PCT protocol

Therapy was carried out under general anesthesia using a biplane flat panel angiographic suite (Artis Q; Siemens). Selective intracranial vessel navigation was carried out upon administration of 3,000 IU heparin intravenously. After femoral arterial access, a 6Fr guide catheter was used for the catheterization of the target artery, and full selective DSA was carried out prior to every treatment.

**Figure 1:**
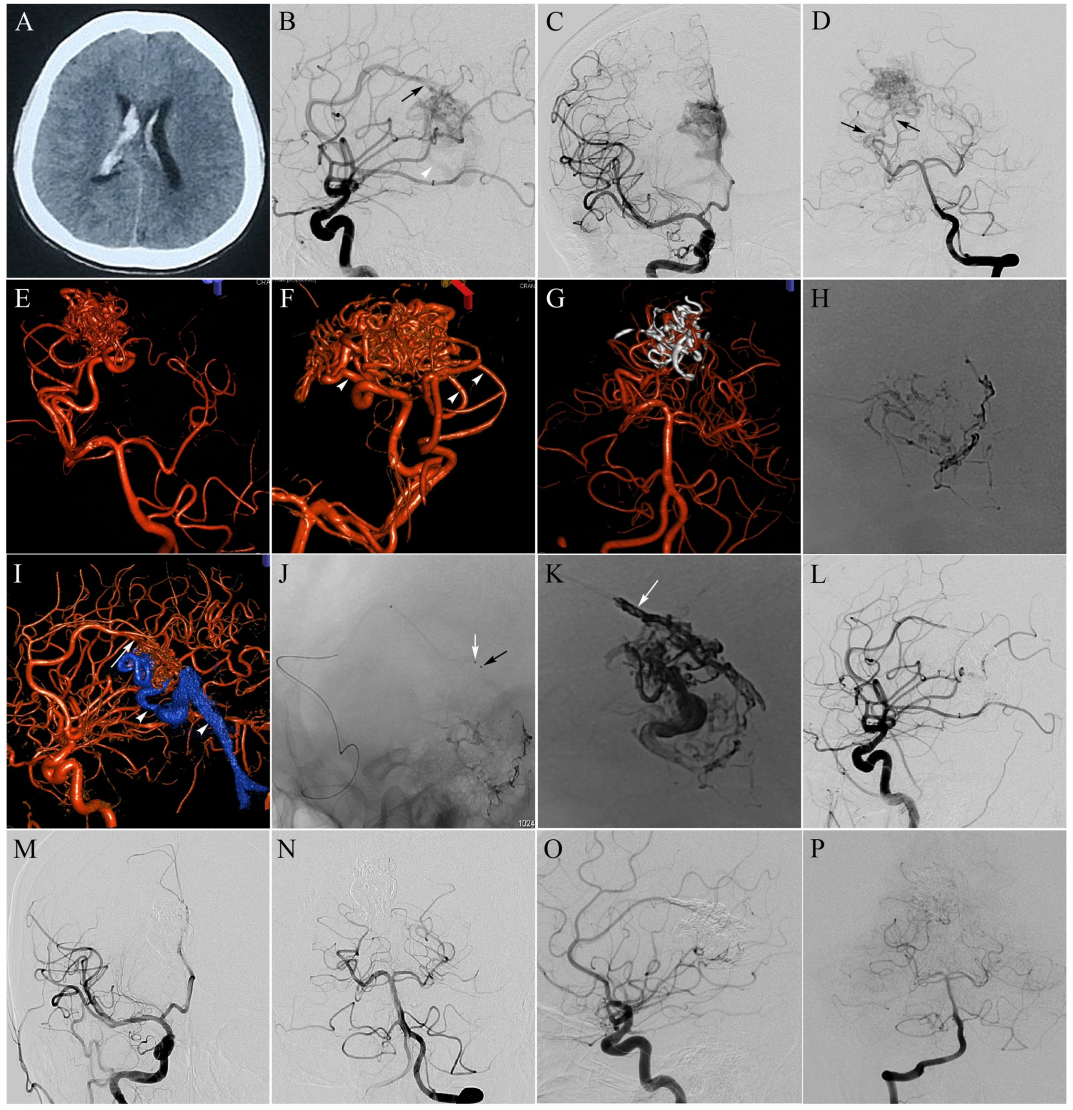
Callosal AVM, Spetzler-Matin grade II. The patient presented with severe headache and meningeal irritation syndrome. **(A)** Axial CT scan at presentation, revealing intraventricular hemorrhage; **(B–D)** Selective right internal carotid artery (ICA) injection [anteroposterior **(B)** and lateral **(C)** views] and selective left vertebral artery (VA) injection [anteroposterior **(D)** view] DSA indicating a 2.8-cm AVM nidus, supplied by a hypertrophied pericallosal artery originating from the right anterior cerebral artery (ACA; black arrow in **B**), as well as 2 main feeders from the right posterior cerebral artery (PCA; black arrows in **D**), and drained by the same venous outlet (white arrowheads in **B** and **I**), into the straight sinus; **(E,F)** Three-dimensional rotational angiography better delineated the arterial feeders from the PCA. Three feeding arteries were selected for preliminary Onyx embolization (white arrowheads in **F**). The goal of this embolization was nidus volume reduction; **(G)** Onyx cast after completing preliminary embolization following 3D reconstruction; **(H)** Single-shot DSA revealing Onyx cast after preliminary embolization; **(I)** Three-dimensional rotational angiography showing enlarged pericallosal artery (white arrow) feeding the nidus; **(J)** Single shot showing the markers of two microcatheter tips of the Marathon (black arrow) and the SL-10 (white arrow) inside the pericallosal artery; **(K)** A plug comprising coils and glue was generated to avoid reflux (white arrow). Single-shot DSA revealing Onyx cast after procedure completion; **(L–N)** Selective right ICA and left VA injection DSA performed immediately postoperatively showing complete nidus exclusion; **(O,P)** At 12-month follow-up, angiography showed persistent total occlusion.

**Figure 2:**
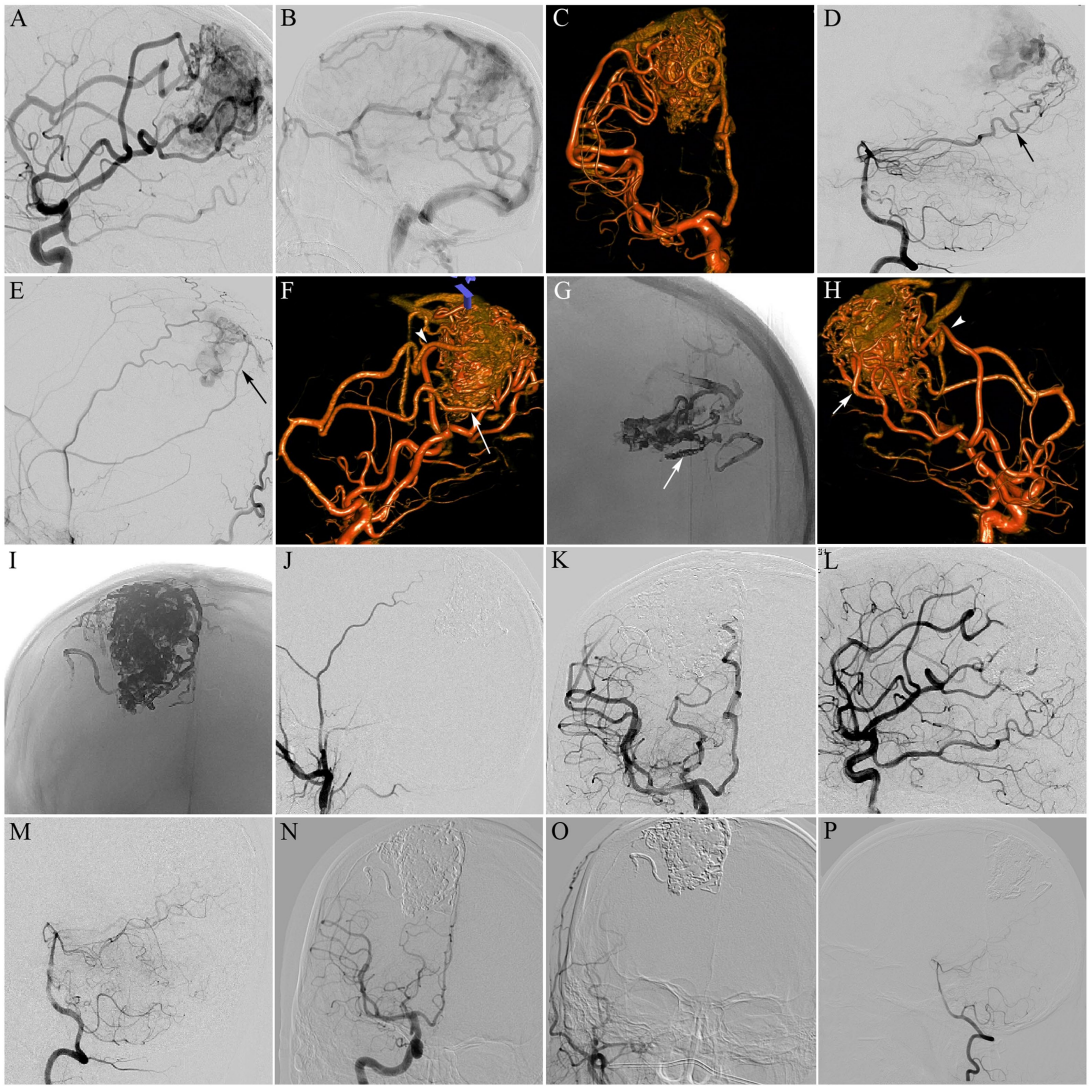
Parietal AVM, Spetzler-Matin grade III. The patient had seizure symptoms. **(A,B)** Selective right internal carotid artery (ICA) injection (lateral views) DSA revealing a 5-cm AVM nidus, supplied by branches from the right anterior (ACA) and middle (MCA) cerebral arteries, and drained by cortical venous to the superior sagittal sinus (SSS); **(C)** Three-dimensional rotational angiography from the right ICA better delineated the angioarchitecture of the AVM; **(D,E)** Selective right vertebral artery (VA) injection [lateral **(D)** view] and selective right external carotid artery (ECA) injection [lateral **(E)** view] DSA showing the nidus, also supplied by the right posterior cerebral artery (PCA; black arrow in **D**) and the right middle meningeal artery (MMA; black arrow in **E**); **(F)** Three-dimensional rotational angiography better delineated enlarged pericallosal artery (white arrow) and enlarged callosomarginal artery (white arrowhead) feeding the nidus. The pericallosal artery was selected for Onyx embolization by the pressure cooker technique; **(G)** A plug of coils and glue was generated to avoid reflux (white arrow). Single-shot DSA revealing Onyx cast after completing the first pressure cooker embolization; **(H)** Three-dimensional rotational angiography showing the nidus, also fed by the superior (white arrowhead) and inferior (white arrow) trunks of the MCA. The inferior trunk of the MCA was selected for the second embolization by the pressure cooker technique; **(I)** Single-shot DSA revealing Onyx cast after completing the second pressure cooker embolization; **(J)** The last embolization was performed through the MMA to occlude the residual nidus. DS angiogram of the right ECA after embolization revealing total obliteration of residual bAVM; **(K–M)** Selective right ICA and left VA injection DSA obtained immediately postoperatively showing complete nidus exclusion; **(N–P)** The 13-month follow-up angiogram confirmed complete bAVM occlusion.

For the PCT, two microcatheters were positioned in the selected feeder. The first microcatheter (Marathon, EV3; Apollo, EV3; Sonic, Balt) was super-selectively advanced to a suitable location of the selected feeder according to the operator’s expectation. To create a plug of coils, the second microcatheter (SL-10, Stryker; Headway-17, Microvention) was navigated just proximal to the tip of Marathon in the same feeder. Coils were deployed as close as possible to the tip of first microcatheter to avoid difficulty in pulling the microcatheter. The number of deployed coils needed to achieve the highest packing density was decided by the feeder’s caliber. The last coil was not detached until the plug was stable enough. This was followed by slow and gradual injection of Onyx into the nidus, with continuous monitoring by biplane subtracted fluoroscopy. Once reflux along the microcatheter was observed, the injection was discontinued for a few seconds and then resumed. This way, a plug was created, forming a permanent barrier to prevent reflux. The arterial pedicles most indicated for PCT were selected by the treating physician depending on each case. Considering the angioarchitecture of the bAVM, a lesion may need two or three sessions of PCT embolization. Right after embolization completion and microcatheter retrieval, global control angiography was performed. The patients were submitted to Dyna CT scanning immediately following the procedure. Heparin was neutralized with protamine. The patients were admitted to the ICU with a 24-h monitoring after the intervention. In this analysis, the patients in the PCT group were all treated with pressure cooker technique at the first procedure. Some patients in PCT group were simultaneously embolized with the CET for complete occlusion of the nidus. Figures 1, 2 show two cases of Onyx embolization with PCT.

For the CET, one microcatheter was advanced in the selected feeder with the tip of microcatheter positioned as close as possible to the nidus of bAVM. No coils were used for creating the anti-reflux plug. We used the “plug-and-push technique,” which consists of fractionated injections of Onyx with pauses of 30–40 s when Onyx reaches the origin of the nidal veins or it reflows into the arterial feeder toward the microcatheter. The microcatheter would be removed after the embolization or when the reflux exceeded 2.0 cm or beyond the determined length. If necessary, a new microcatheter was positioned into another pedicle and embolization was continued in the same way.

### Study variables and definitions

The Spetzler-Martin grading scale included bAVM size, venous draining pattern, and eloquence (12). Deep location was defined as any involvement of the corpus callosum, thalamus, basal ganglia, brainstem, cerebellar peduncles, or deep cerebellar nuclei (13). Perforating artery supply included lateral lenticulostriate arteries from the middle cerebral artery (MCA)’s M1 segment, medial lenticulostriate arteries from the anterior cerebral artery (ACA)’s A1 segment, anterior and posterior choroidal arteries, thalamic perforators from the posterior communicating artery and posterior cerebral artery (PCA)’s P1 segment, and brainstem perforators from the basilar trunk and vertebral arteries (14). Single feeding artery was defined as ACA, MCA, PCA, superior cerebellar artery (SCA), anterior inferior cerebellar artery (AICA), or posterior inferior cerebellar artery (PICA). Compact and diffuse bAVM morphologies were assessed by preoperative DSA, and CT and MRI were performed for identifying intervening brain parenchyma within the nidus. Hemorrhage was evidenced by CT or MRI signals. The modified Rankin Scale (mRS) was employed for grading functional outcomes [mRS score < 3 (favorable) vs. mRS score ≥ 3 (unfavorable)]. Radiological obliteration was reflected by the complete absence of the nidus and early draining veins as detected by DSA.

### Statistical analysis

Data were analyzed with SPSS 23 (SPSS, USA). Continuous and categorical variables were presented as mean ± SD and frequency or percentage, respectively, and compared by the Student t-test and the chi-square or Fisher’s exact test, respectively. Multivariable logistic regression analysis of variables with *p* < 0.20 in univariable analysis was carried out to identify factors independently affecting complete bAVM obliteration in the PCT group. *p* < 0.05 was deemed to reflect statistical significance. Odds ratio (OR) and 95% confidence interval (CI) were determined for each variable included in the multivariate model.

## Results

### Patient and bAVM characteristics

There were 46 and 79 in the PCT and CET groups, respectively. All patient-, lesion-, and outcome-related data about this single procedure were collected from the database and analyzed. Table 1 shows patient demographic and bAVM characteristics in the PCT and CET groups. The two patient groups were similar with respect to sex, age, clinical presentation, hemorrhage, Spetzler-Martin grade, and preoperative mRS score.

**Table 1:** Patient and bAVM characteristics.

Variable	Total	PCT	CET	*p*-Value*
No. of patients	125	46	79	
Sex				0.85
Male	81 (65)	29 (63)	52 (66)	
Female	44 (35)	17 (37)	27 (34)	
Mean age at surgery, yrs.	31.5 ± 16.7	31.6 ± 14.7	31.4 ± 17.8	0.96
Clinical presentation				
Seizure	13 (10)	7 (15)	6 (8)	0.23
Headache	21 (17)	9 (20)	12 (15)	0.62
Neurological deficits	1 (0.8)	0 (0)	1 (1)	1.00
Incidental	19 (15)	9 (20)	10 (13)	0.31
Hemorrhage	72 (58)	22 (48)	50 (63)	0.13
Spetzler-Martin grade				0.58
I	34 (27)	11 (24)	23 (29)	
II	47 (38)	17 (37)	30 (38)	
III	35 (28)	14 (30)	21 (27)	
IV	7 (6)	4 (9)	3 (4)	
V	2 (2)	0 (0)	2 (3)	
Preop mRS score				1.00
<3	116 (93)	43 (93)	73 (92)	
≥3	9 (7)	3 (7)	6 (8)	

PCT, pressure cooker technique; CET, conventional embolization technique.

* PCT group versus CET group.

Values indicate the number of patients (%) unless otherwise indicated. Other data are mean ± SD.

### Procedure parameters and procedure-related complications

Table 2 lists procedural characteristics and complications for the 125 patients. Totally 232 arterial pedicles (103 and 129 in the PCT and CET groups, respectively) were subjected to embolization. Of the 103 arterial pedicles in the PCT group, 60 were treated by the PCT, and the details are summarized in Table 2. Procedure-related complications, including intra-operative hemorrhage, post-operative hemorrhage, and cerebral ischemia, occurred in 6 (13%) patients administered the PCT versus 8 (10%) in the CET group. The overall complication rates were similar in both groups (*p* = 0.77). In these cases, hemorrhagic events caused deterioration in mRS scores following embolization in four patients of the PCT group (4/46 patients, 9%) versus two patients of the CET group (2/79 patients, 3%). The rate was non-significantly lower in the CET group compared with the PCT group (*p* = 0.19). In these six patients, 3 (2 and 1 in the PCT and CET groups, respectively) had a change of three points in mRS score, and the remaining three patients (2 and 1 in the PCT and CET groups, respectively) had a change of ≥4 points. One patient each in the PCT and CET groups were treated by external ventricle drainage after embolization. Three and one patients in the PCT and CET groups, respectively, were treated by emergency hematoma evacuation after embolization. In addition, two and three patients in the PCT and CET groups, respectively, had cerebral ischemia after embolization. Neurological deficits occurred in one patient of the CET group. Besides, one patient with brainstem-fourth ventricle AVM in the CET group died of medullary infarction after embolization. The remaining three patients with cerebral ischemia were conservatively treated with medications and their neurologic status did not change.

**Table 2:** Characteristics and outcomes of the embolization procedure.

Variable	Total	PCT	CET	*p*-Value*
No. of embolized pedicles	232	103	129	
No. of pedicles treated with PCT	60	60	0	
No. of patients	125	46	79	
One pedicle treated with PCT	35 (76)	35 (76)	0	
Two pedicles treated with PCT	8 (17)	8 (17)	0	
Three pedicles treated with PCT	3 (7)	3 (7)	0	
Angiographic outcomes				
Complete obliteration	61 (49)	28 (61)	33 (42)	**0.04**
Partial obliteration	64 (51)	18 (39)	46 (58)	
Spetzler-Martin grade ≤ II				
Complete obliteration	52 (64)	24 (86)	28 (53)	**0.0036**
Partial obliteration	29 (36)	4 (14)	25 (47)	
Spetzler-Martin grade ≥ III				
Complete obliteration	9 (20)	4 (22)	5 (19)	1.00
Partial obliteration	35 (80)	14 (78)	21 (81)	
Procedure-related complications	14 (11)	6 (13)	8 (10)	0.77
Intraop ICH	3 (2)	0 (0)	3 (4)	
Postop ICH	6 (5)	4 (9)	2 (3)	
Cerebral ischemia	5 (4)	2 (4)	3 (4)	
mRS score at 30 days				1.00
<3	112 (90)	41 (89)	71 (90)	
≥3	13 (10)	5 (11)	8 (10)	

PCT, pressure cooker technique; CET, conventional embolization technique; ICH = intracerebral hemorrhage.

* PCT group versus CET group.

Values indicate the number of patients or pedicles (%) unless otherwise indicated. Other data are mean ± SD. Bold indicates statistical significance.

### Angiographic outcomes

The primary aim of the present work was to evaluate angiographic bAVM obliteration immediately postembolization (Table 2). In the first procedure, complete obliteration immediately after embolization was detected in 61 and 42% of patients in the PCT and CET groups, respectively, indicating markedly elevated rate in the PCT group (*p* = 0.04). In subgroup analysis, the rate of immediate complete obliteration was starkly elevated in PCT group patients with Spetzler-Martin grade I/II bAVMs, with 24/28 (86%) and 28/53 (53%) cases in the PCT and CET groups, respectively (*p* = 0.0036). However, in patients with Spetzler-Martin grade III or higher bAVMs, immediate complete obliteration had similar rates in both groups, with 4/18 (22%) and 5/26 (19%) in the PCT and CET groups, respectively (*p* = 1.00).

### Univariate and multiple logistic regression analyses

In univariate analysis, five variables (nidus size, diffuse nidus, deep location, single feeding artery and concurrent AVF) had *p*-values <0.20 and were entered into a multivariate logistic regression analysis to determine factors independently affecting complete bAVM obliteration in the PCT group (Table 3). In multivariate logistic regression analysis, nidus size >3 cm (OR = 8.826, 95% CI: 1.250–62.312; *p* = 0.03) and deep location (OR = 8.576, 95% CI: 1.480–49.690; *p* = 0.02) were significant predictive factors of incomplete obliteration in the PCT group (Table 3).

**Table 3:** Univariate and multivariate analyses for predictors of incomplete bAVM obliteration in the PCT group.

Covariates	Univariate			Multivariate		
	OR	95% CI	*P*-value	OR	95% CI	*P*-value
Sex	2.500	0.727–8.601	0.21			
Nidus size >3 cm	6.000	1.633–22.040	0.007	8.826	1.250–62.312	**0.03**
Deep venous drainage	0.524	0.146–1.878	0.35			
Perforating artery supply	0.565	0.137–2.325	0.48			
Hemorrhage	0.715	0.241–2.120	0.59			
Diffuse nidus	0.333	0.079–1.411	0.16	0.221	0.021–2.369	0.21
Deep location	4.800	1.173–19.640	0.03	8.576	1.480–49.690	**0.02**
Single feeding artery	4.018	1.117–14.460	0.04	3.440	0.724–16.356	0.12
Accompanied with AVF	0.269	0.044–1.658	0.19	5.242	0.422–65.062	0.19

AVF, arterio-venous fistula; CI, confidence interval; OR, odds ratio.

Bold indicates statistical significance.

### Follow-Up

In these cases, with the results that bAVMs were completely occluded, bAVM recurrence occurred in one patient of the PCT group (1/28, 4%) versus three in the CET group (3/33, 9%), indicating a non-significantly lower rate in the PCT group (*p* = 0.62). Mean time to follow-up was 11.4 months (median of 12 months, ranging from 5 to 21 months). In those cases in which the bAVM was not completely occluded, patients underwent complementary treatment including staged embolization, neurosurgery and radiosurgery. The concrete techniques applied and the results of these complementary treatments were not available.

## Discussion

With the advent of Onyx, embolization is gradually becoming a curative therapeutic option for complete occlusion of bAVMs (15). Onyx utilization has resulted in complete obliteration rate increases to up to 51% for all bAVMs and 96% for superficial, small-to-medium-sized bAVMs (3, 4, 6, 7). Onyx has increased the potential to cure bAVMs, hence many operators prefer achieving complete embolization in the fewest possible number of sessions. Single-stage embolization may be safer and more efficacious than multiple-stage embolizations, because it reduces the overall time under treatment, thereby curtailing the intertreatment risk and the potential for bAVM collateral vessel reconstitution. With the evolution of endovascular equipment and techniques, single-stage embolization of bAVMs with an intent to cure has become therapeutic goal in our center. However, controlling the direction of Onyx flow and directing reflux proximally remains a challenge. To overcome this problem, the PCT was introduced by Chapot et al. (8). Daniel et al. presented the modified PCT to render reflux control easier and more reproducible, thereby simplifying the procedure (10). Lately, proximal coil protection and a balloon or microballoon were used to enhance bAVM embolization. Though the materials were different, the mechanisms were comparable to those of the PCT technique.

This retrospective analysis focused on the safety and efficacy of the PCT compared with the CET in two bAVM groups similar in baseline characteristics. According to our findings, the PCT had higher efficacy compared with the conventional technique in transarterial embolization for bAVMs. In addition, the PCT and CET groups had comparable complication rates.

As reported previously, the PCT is safe and effective in bAVM treatment, with more efficient and controlled Onyx injection, reduced reflux risk and lower fluoroscopy times. However, the data were mostly limited to case reports. In our comparative study data, PCT application resulted in elevated rate of immediate complete obliteration compared with the use of the conventional technique within a single session (61 and 42% in the PCT and CET groups, respectively; *p* = 0.04). The PCT is particularly useful for Spetzler-Martin Grade I or II bAVMs (86 and 53% in the PCT and CET groups, respectively; *p* = 0.0036). However, for Spetzler-Martin Grade III or higher bAVMs, the PCT did not show the same benefits (22 and 19% in the PCT and CET groups, respectively; *p* = 1.00). Therefore, use of the PCT considered on an individual basis may be more meaningful. For low-grade bAVMs (Spetzler-Martin Grade I/II), the PCT appears to be a highly effective means for single-stage embolization. For high-grade bAVMs (Spetzler-Martin Grade III or higher), a gradual exclusion of the nidus with staged procedures is needed. In our experience, additionally, while evaluating an bAVM for the PCT, injection of a relatively large amount of Onyx through a single feeder and opening of intra-nidal channels between different compartments should be considered. Therefore, the main parameter of this method is feeder size. In case of large readily accessible vessels feeding the nidus, which could accommodate the stiff plug (coils + Onyx), complete obliteration was achievable, sometimes *via* a single feeder. Therefore, the PCT could be highly personalized and relies on the angioarchitectural features of bAVMs. In the present approach, mastering the technique and the decision about the optimal embolization method were critical determinants of good outcome. In these cases, although there was no procedural complications related to retrieval of the microcatheter after Onyx injection, we still recommend using detachable tip microcatheter in PCT. Detachable tip microcatheter theoretically reduce the risk during microcatheter removal.

The traditional method, which relies on the formation of a ‘proximal plug’, is time-consuming and unpredictable, resulting in a long and slow Onyx penetration inside the nidus, which subsequently prolongs the procedure time and increases fluoroscopy time. Multiple reports have shown a dual-lumen balloon microcatheter could be utilized in lieu of the ‘proximal plug’ of Onyx, improving the success of the endovascular treatment of bAVMs (16–18). Total malformation occlusion was obtained in 15–61% of bAVMs. In our hands, however, balloon navigability showed inferiority to flow-guided microcatheters. Sometimes, the navigability of the balloon microcatheter into the target vessel is hampered by severe tortuosity of the target vessel. Besides, handling of a balloon microcatheter is more delicate because the pedicle could rupture *via* balloon inflation in the very small artery.

In this analysis, the patients in the PCT group were all treated with pressure cooker technique; if supplementary embolization was necessary, some patients were treated with the CET additionally for complete occlusion of the nidus. The patients in CET group were only treated with conventional embolization technique. At the beginning (2019), the CET was used, and after we developed the PCT, since 2020, these 2 techniques have been alternately used in the patients. Although the operator could directly compare the two different approaches, potential differences in treatment epochs between the PCT group vs. CET group may introduce bias into the study. Therefore, further studies are needed to explore further the usefulness of the technique in bAVMs.

Multivariate analysis revealed that factors associated with incomplete obliteration in the PCT group were nidus size >3 cm and deep location. Similar findings were published by Jordan and collaborators who demonstrated a nidus size of 30–60 mm is associated with incomplete obliteration (19). Van Rooij et al. reported a nidus size of 10–30 mm as a factor associated with complete obliteration (6). These studies corroborated our results, in which larger size decreased the odds of total occlusion. Besides, deep-seated bAVMs are very challenging to manage by invasive treatment tools. Transarterial embolization for deep-seated bAVMs is rarely reported. Relatively small case series (20–22) have reported total occlusion rates of 2.6–43%, often applying stereotactic radiosurgery as an adjunct approach for treating residual nidus. Given that establishing arterial access *via* the small perforating feeders is challenging and has high complication risk, endovascular tools are seldom curative and often utilized as an adjunct tool for a definitive therapy (23). Besides, such lesions are very demanding for microsurgery due to their intrinsic association with major neural structures and reduced surgical exposure (24). Considering these technical challenges, transvenous embolization or multimodality therapy may be the future direction of therapy (25, 26).

As shown above, the PCT and CET groups showed comparable rates of procedure-related complications. Our total complication rates in both groups (11% morbidity and 0.8% mortality) were lower than those reported in a systematic review of endovascular embolization of bAVMs (24.1% morbidity and 1.5% mortality) (15). The reason may be that 65% (81 of 125) of bAVMs in this study were Spetzler-Martin grade I-II. Another concern was bAVM recurrence after initial complete angiographic obliteration in the current study. There are two proposed mechanisms of bAVM recurrence after embolization. First, recurrence is reflected by residual nidus, which is angiographically occult because of obscuration from vessel spasm, transient thrombosis or mass effect from adjacent hematoma/cerebral edema in the early postoperative period. Further resolution would promote the recanalization of the residual shunt. Secondly, “hidden compartments,” i.e., unfilled bAVM regions surround the active nidus due to low or no flow from internal steal developing from hemodynamic alterations after embolization (27).

## Limitations

We acknowledge that the current study has limitations. First, the number of bAVMs treated with the PCT was small (46 patients), so large-sample trials are warranted to confirm the above findings. Secondly, selection bias was inevitable in this retrospective study performed in a single center. The heterogeneous nature of the 2 groups of patients causes their comparison to be less reliable. Many factors contributed to the patient population nonhomogeneous. For instance, the same microcatheter was not used for all treatments; the use of PCT relied on the discretion of the operator; some patients in this series received radiosurgery and/or embolization in other hospitals before this procedure. Finally, the subset of patients with partial bAVM occlusion lacks adequate follow-up, which might reveal unexpected findings and change the overall cure rate of the PCT.

## Conclusion

The PCT seems to yield better angiographic results for bAVMs and is especially suitable for low-grade bAVMs. It could increase the immediate cure rate of bAVMs without increasing procedure-related complications.

## Data availability statement

The original contributions presented in the study are included in the article/supplementary material, further inquiries can be directed to the corresponding authors.

## Ethics statement

The studies involving human participants were reviewed and approved by Institutional human research and ethics committee of Tangdu Hospital. The patients/participants provided their written informed consent to participate in this study.

## Author contributions

DL acquired and analyzed data, and wrote the first draft of the manuscript. YL and ZY performed imaging analyses and acquired related data. TM, NW, and XL acquired clinical data. ZZ, WF, and LC contributed to the database. TZ and JD contributed to the conception and design of the study and reviewed the manuscript. All authors contributed to the article and approved the submitted version.

## Conflict of interest

The authors declare that the research was conducted in the absence of any commercial or financial relationships that could be construed as a potential conflict of interest.

## Publisher’s note

All claims expressed in this article are solely those of the authors and do not necessarily represent those of their affiliated organizations, or those of the publisher, the editors and the reviewers. Any product that may be evaluated in this article, or claim that may be made by its manufacturer, is not guaranteed or endorsed by the publisher.
